# Supplementary material for: Predicting Axillary Lymph Node Metastasis in Early Breast Cancer Using Deep Learning on Primary Tumor Biopsy Slides
Source: Front Oncol. 2021 Oct 14;11:759007. doi: 10.3389/fonc.2021.759007 (PMC8551965; doi:10.3389/fonc.2021.759007)
Supplement: Supplementary file 1 [file DataSheet_1.docx]

Filename: “Software”

Description: The executive program (.exe file) is used to predict ALN status with “demo data” we provided, and “checkpoints” contains the parameters of the DL-CNB+C model that will be loaded by the executive program.

Please download our demo software “Software” using the following address：

https://drive.google.com/drive/folders/1ItKCldu8vbHhbZvhXic-11Ei-NVGBZU2?usp=sharing
