# Supplementary material for: Predicting Axillary Lymph Node Metastasis in Early Breast Cancer Using Deep Learning on Primary Tumor Biopsy Slides
Source: Front Oncol. 2021 Oct 14;11:759007. doi: 10.3389/fonc.2021.759007 (PMC8551965; doi:10.3389/fonc.2021.759007)
Supplement: Supplementary file 7 [file Image_1.pdf]

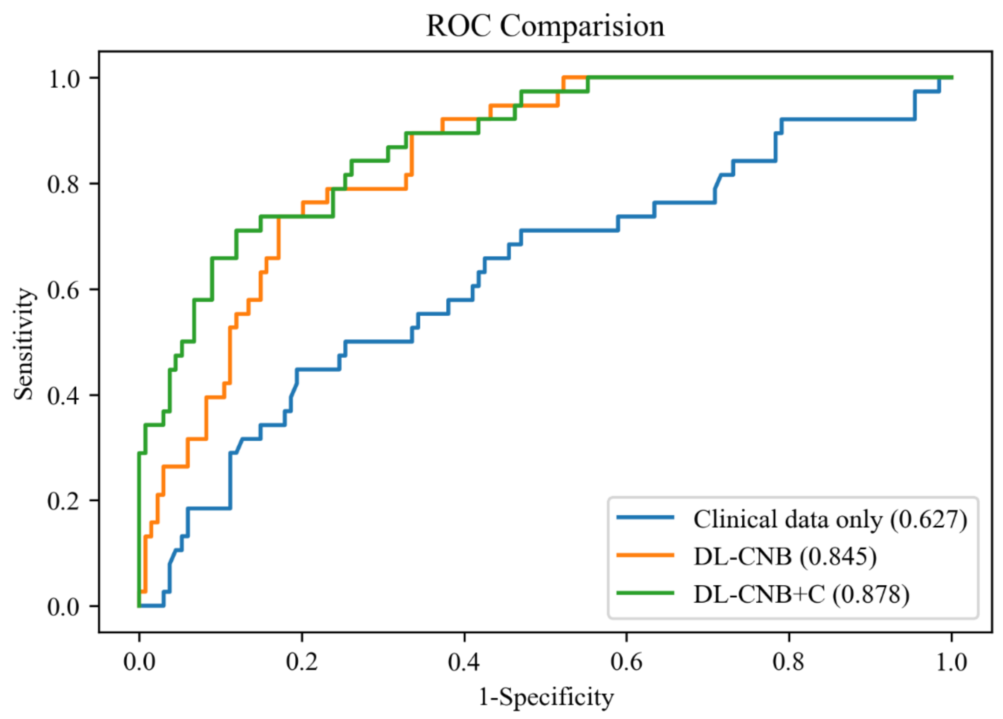

Figure 1. Comparison of receiver operating characteristic (ROC) curves between different models for predicting disease-free axilla (N0) and low metastatic burden of axillary disease (N+(1-2)). Numbers in parentheses are AUCs.
