# Supplementary material for: Predicting Axillary Lymph Node Metastasis in Early Breast Cancer Using Deep Learning on Primary Tumor Biopsy Slides
Source: Front Oncol. 2021 Oct 14;11:759007. doi: 10.3389/fonc.2021.759007 (PMC8551965; doi:10.3389/fonc.2021.759007)
Supplement: Supplementary file 9 [file Table_1.docx]

| **Table 1 The performance comparison of different base models in prediction of ALN status (N0 vs. N(+)).** | | | | | | | |
| --- | --- | --- | --- | --- | --- | --- | --- |
| **Base models** |  | **AUC** | **ACC (%)** | **SENS (%)** | **SPEC (%)** | **PPV (%)** | **NPV (%)** |
| AlexNet | T | 0.909 [0.884, 0.930] | 82.70 [79.51, 85.57] | 88.33 [83.58, 92.11] | 79.23 [74.86, 83.15] | 72.35 [68.20, 76.16] | 91.69 [88.59, 94.01] |
|  | V | 0.764 [0.700, 0.819] | 65.71 [58.87, 72.11] | 89.87 [81.02, 95.53] | 51.15 [42.26, 59.97] | 52.59 [47.84, 57.30] | 89.33 [80.96, 94.28] |
|  | I-T | 0.780 [0.719, 0.833] | 73.39 [67.01, 79.13] | 83.33 [73.62, 90.58] | 67.16 [58.53, 75.03] | 61.40 [55.08, 67.36] | 86.54 [79.71, 91.32] |
| ResNet50 | T | 0.912 [0.887, 0.933] | 85.71 [82.74, 88.35] | 85.83 [80.77, 89.99] | 85.64 [81.76, 88.97] | 78.63 [74.17, 82.50] | 90.76 [87.77, 93.08] |
|  | V | 0.644 [0.575, 0.709] | 59.52 [52.55, 66.22] | 70.89 [59.58, 80.57] | 52.67 [43.77, 61.45] | 47.46 [41.80, 53.19] | 75.00 [67.22, 81.44] |
|  | I-T | 0.607 [0.539, 0.673] | 58.72 [51.87, 65.32] | 66.67 [55.54, 76.58] | 53.73 [44.92, 62.38] | 47.46 [41.61, 53.37] | 72.00 [64.65, 78.33] |
| DenseNet121 | T | 0.967 [0.949, 0.979] | 89.84 [87.21, 92.09] | 95.83 [92.47, 97.98] | 86.15 [82.32, 89.42] | 80.99 [76.85, 84.53] | 97.11 [94.82, 98.41] |
|  | V | 0.714 [0.648, 0.774] | 68.57 [61.82, 74.79] | 73.42 [62.28, 82.73] | 65.65 [56.85, 73.72] | 56.31 [49.56, 62.84] | 80.37 [73.56, 85.77] |
|  | I-T | 0.739 [0.675, 0.796] | 69.27 [62.68, 75.32] | 85.71 [76.38, 92.39] | 58.96 [50.13, 67.37] | 56.69 [51.21, 62.02] | 86.81 [79.28, 91.89] |
| Inception-v3 | T | 0.968 [0.951, 0.980] | 91.75 [89.32, 93.77] | 95.42 [91.95, 97.69] | 89.49 [86.01, 92.35] | 84.81 [80.68, 88.20] | 96.94 [94.68, 98.26] |
|  | V | 0.753 [0.689, 0.810] | 70.48 [63.81, 76.55] | 67.09 [55.60, 77.25] | 72.52 [64.04, 79.95] | 59.55 [51.71, 66.93] | 78.51 [72.39, 83.59] |
|  | I-T | 0.762 [0.700, 0.817] | 71.10 [64.59, 77.02] | 85.71 [76.38, 92.39] | 61.94 [53.16, 70.18] | 58.54 [52.79, 64.06] | 87.37 [80.12, 92.23] |
| VGG16_BN | T | 0.901 [0.875, 0.923] | 80.32 [76.99, 83.35] | 94.17 [90.41, 96.77] | 71.79 [67.05, 76.21] | 67.26 [63.61, 70.71] | 95.24 [92.30, 97.09] |
|  | V | 0.808 [0.748, 0.859] | 72.86 [66.31, 78.75] | 77.22 [66.40, 85.90] | 70.23 [61.62, 77.90] | 61.00 [53.95, 67.62] | 83.64 [77.04, 88.62] |
|  | I-T | 0.816 [0.758, 0.865] | 74.77 [68.46, 80.39] | 80.95 [70.92, 88.70] | 70.90 [62.43, 78.42] | 63.55 [56.76, 69.84] | 85.59 [79.04, 90.34] |
| VGG16_BN+C | T | 0.878 [0.622, 0.698] | 76.51 [73.00, 79.77] | 93.33 [89.40, 96.14] | 66.15 [61.22, 70.84] | 62.92 [59.53, 66.19] | 94.16 [90.90, 96.30] |
|  | V | 0.823 [0.765, 0.872] | 75.71 [69.34, 81.35] | 74.68 [63.64, 83.80] | 76.34 [68.12, 83.32] | 65.56 [57.69, 72.65] | 83.33 [77.19, 88.08] |
|  | I-T | 0.831 [0.775, 0.878] | 75.69 [69.44, 81.23] | 89.29 [80.63, 94.98] | 67.16 [58.53, 75.03] | 63.03 [56.96, 68.71] | 90.91 [84.21, 94.94] |
| 95% confidence intervals are included in brackets.  *AUC* area under the receiver operating characteristic curve, *ACC* accuracy, *SENS* sensitivity, *SPEC* specificity, *PPV* positive predict value, *NPV* negative predict value.  *T* training cohort (n = 630), *V* validation cohort (n = 210), *I–T* independent test cohort (n = 218). | | | | | | | |
