# Supplementary material for: Predicting Axillary Lymph Node Metastasis in Early Breast Cancer Using Deep Learning on Primary Tumor Biopsy Slides
Source: Front Oncol. 2021 Oct 14;11:759007. doi: 10.3389/fonc.2021.759007 (PMC8551965; doi:10.3389/fonc.2021.759007)
Supplement: Supplementary file 10 [file Table_2.docx]

| **Table 2 The detailed parameters of VGG16_BN.** | | | | | | |
| --- | --- | --- | --- | --- | --- | --- |
| **Layer name** | **Input channels** | **Output channels** | **Kernel size** | **Stride** | **Padding** | **Output size** |
| basic block×2 | 3 | 64 | 3 | 1 | 1 | [64, 256, 256] |
| max pooling layer |  |  | 2 | 2 | 0 | [64, 128, 128] |
| basic block×2 | 64 | 128 | 3 | 1 | 1 | [128, 128, 128] |
| max pooling layer |  |  | 2 | 2 | 0 | [128, 64, 64] |
| basic block×3 | 128 | 256 | 3 | 1 | 1 | [256, 64, 64] |
| max pooling layer |  |  | 2 | 2 | 0 | [256, 32, 32] |
| basic block×3 | 256 | 512 | 3 | 1 | 1 | [512, 32, 32] |
| max pooling layer |  |  | 2 | 2 | 0 | [512, 16, 16] |
| basic block×3 | 512 | 512 | 3 | 1 | 1 | [512, 16, 16] |
| max pooling layer |  |  | 2 | 2 | 0 | [512, 8, 8] |
| adaptive average pooling layer |  |  |  |  |  | [512, 7, 7] |
| The basic block was cascade by convolution layer, batch normalization layer, and ReLU.  The input size of the model was [3, 256, 256], which followed the format of [channel, height, width]. | | | | | | |
