# Supplementary material for: Predicting Axillary Lymph Node Metastasis in Early Breast Cancer Using Deep Learning on Primary Tumor Biopsy Slides
Source: Front Oncol. 2021 Oct 14;11:759007. doi: 10.3389/fonc.2021.759007 (PMC8551965; doi:10.3389/fonc.2021.759007)
Supplement: Supplementary file 11 [file Table_3.docx]

| **Table 3 The performance in prediction of ALN status (N0 vs. N+(1-2)).** | | | | | | | |
| --- | --- | --- | --- | --- | --- | --- | --- |
| **Methods** |  | **AUC** | **ACC (%)** | **SENS (%)** | **SPEC (%)** | **PPV (%)** | **NPV (%)** |
| Clinical data only | T | 0.638 [0.595, 0.679] | 61.00 [56.65, 65.23] | 65.62 [56.72, 73.79] | 59.49 [54.43, 64.40] | 34.71 [30.88, 38.75] | 84.06 [80.37, 87.16] |
|  | V | 0.677 [0.602, 0.745] | 74.29 [67.15, 80.58] | 45.45 [30.39, 61.15] | 83.97 [76.55, 89.79] | 48.78 [36.42, 61.29] | 82.09 [77.60, 85.84] |
|  | I-T | 0.627^a, b^ [0.551, 0.700] | 72.67 [65.37, 79.18] | 44.74 [28.62, 61.70] | 80.60 [72.88, 86.92] | 39.53 [28.52, 51.73] | 83.72 [79.24, 87.39] |
| DL-CNB model | T | 0.912 [0.884, 0.935] | 82.24 [78.67, 85.44] | 97.66 [93.30, 99.51] | 77.18 [72.69, 81.25] | 58.41 [53.87, 62.81] | 99.01 [97.04, 99.68] |
|  | V | 0.756 [0.685, 0.817] | 59.43 [51.76, 66.77] | 97.73 [87.98, 99.94] | 46.56 [37.81, 55.48] | 38.05 [34.22, 42.04] | 98.39 [89.70, 99.77] |
|  | I-T | 0.845^c^ [0.782, 0.895] | 80.23 [73.49, 85.90] | 73.68 [56.90, 86.60] | 82.09 [74.53, 88.17] | 53.85 [43.66, 63.72] | 91.67 [86.53, 94.96] |
| DL-CNB+C model | T | 0.936 [0.911, 0.955] | 84.17 [80.74, 87.21] | 95.31 [90.08, 98.26] | 80.51 [76.23, 84.33] | 61.62 [56.66, 66.34] | 98.12 [95.99, 99.13] |
|  | V | 0.789 [0.721, 0.847] | 66.29 [58.76, 73.24] | 84.09 [69.93, 93.36] | 60.31 [51.39, 68.74] | 41.57 [35.72, 47.67] | 91.86 [84.94, 95.76] |
|  | I-T | 0.878 [0.819, 0.923] | 84.30 [77.99, 89.39] | 71.05 [54.10, 84.58] | 88.06 [81.33, 93.02] | 62.79 [50.52, 73.61] | 91.47 [86.65, 94.66] |
| 95% confidence intervals are included in brackets.  *AUC* area under the receiver operating characteristic curve, *ACC* accuracy, *SENS* sensitivity, *SPEC* specificity, *PPV* positive predict value, *NPV* negative predict value.  *T* training cohort (n = 518), *V* validation cohort (n = 175), *I–T* independent test cohort (n = 172).  ^a^Indicates *p* = 0.0004, Delong et al. in comparison with DL-CNB model in independent test cohort.  ^b^Indicates *p* < 0.0001, Delong et al. in comparison with DL-CNB+C model in independent test cohort.  ^c^Indicates *p* = 0.1148, Delong et al. in comparison with DL-CNB+C model in independent test cohort. | | | | | | | |
