# Supplementary material for: Predicting Axillary Lymph Node Metastasis in Early Breast Cancer Using Deep Learning on Primary Tumor Biopsy Slides
Source: Front Oncol. 2021 Oct 14;11:759007. doi: 10.3389/fonc.2021.759007 (PMC8551965; doi:10.3389/fonc.2021.759007)
Supplement: Supplementary file 12 [file Table_4.docx]

| **Table 4 The performance in prediction of ALN status (N0 vs. N+(≥3)).** | | | | | | | |
| --- | --- | --- | --- | --- | --- | --- | --- |
| **Methods** |  | **AUC** | **ACC (%)** | **SENS (%)** | **SPEC (%)** | **PPV (%)** | **NPV (%)** |
| Clinical data only | T | 0.680 [0.638, 0.721] | 66.67 [62.39, 70.75] | 65.83 [56.62, 74.24] | 66.92 [62.01, 71.58] | 37.98 [33.59, 42.58] | 86.42 [83.10, 89.18] |
|  | V | 0.748 [0.675, 0.813] | 71.52 [63.98, 78.26] | 76.47 [58.83, 89.25] | 70.23 [61.62, 77.90] | 40.00 [32.57, 47.92] | 92.00 [86.13, 95.51] |
|  | I-T | 0.629^a, b^ [0.553, 0.701] | 69.36 [61.92, 76.14] | 53.85 [37.18, 69.91] | 73.88 [65.59, 81.08] | 37.50 [28.54, 47.40] | 84.62 [79.43, 88.68] |
| DL-CNB model | T | 0.906 [0.877, 0.930] | 81.57 [77.93, 84.84] | 93.33 [87.29, 97.08] | 77.95 [73.50, 81.97] | 56.57 [51.79, 61.23] | 97.44 [95.10, 98.67] |
|  | V | 0.755 [0.682, 0.819] | 64.24 [56.42, 71.54] | 91.18 [76.32, 98.14] | 57.25 [48.32, 65.85] | 35.63 [30.67, 40.92] | 96.15 [89.36, 98.67] |
|  | I-T | 0.837^c^ [0.773, 0.888] | 69.94 [62.52, 76.67] | 92.31 [79.13, 98.38] | 63.43 [54.68, 71.58] | 42.35 [36.61, 48.31] | 96.59 [90.46, 98.83] |
| DL-CNB+C model | T | 0.918 [0.891, 0.940] | 82.16 [78.55, 85.38] | 91.67 [85.21, 95.93] | 79.23 [74.86, 83.15] | 57.59 [52.62, 62.42] | 96.87 [94.45, 98.25] |
|  | V | 0.761 [0.689, 0.824] | 66.06 [58.29, 73.24] | 79.41 [62.10, 91.30] | 62.60 [53.72, 70.89] | 35.53 [29.40, 42.16] | 92.13 [85.66, 95.83] |
|  | I-T | 0.838 [0.774, 0.889] | 71.10 [63.73, 77.73] | 89.74 [75.78, 97.13] | 65.67 [56.98, 73.65] | 43.21 [37.04, 49.60] | 95.65 [89.61, 98.25] |
| 95% confidence intervals are included in brackets.  *AUC* area under the receiver operating characteristic curve, *ACC* accuracy, *SENS* sensitivity, *SPEC* specificity, *PPV* positive predict value, *NPV* negative predict value.  *T* training cohort (n = 510), *V* validation cohort (n = 165), *I–T* independent test cohort (n = 173).  ^a^Indicates *p* = 0.0005, Delong et al. in comparison with DL-CNB model in independent test cohort.  ^b^Indicates *p* < 0.0001, Delong et al. in comparison with DL-CNB+C model in independent test cohort.  ^c^Indicates *p* = 0.9689, Delong et al. in comparison with DL-CNB+C model in independent test cohort. | | | | | | | |
