# Supplementary material for: Predicting Axillary Lymph Node Metastasis in Early Breast Cancer Using Deep Learning on Primary Tumor Biopsy Slides
Source: Front Oncol. 2021 Oct 14;11:759007. doi: 10.3389/fonc.2021.759007 (PMC8551965; doi:10.3389/fonc.2021.759007)
Supplement: Supplementary file 13 [file Table_5.docx]

| **Table 5 The subgroup performance in prediction of ALN status by DL-CNB+C model (N0 vs. N(+)).** | | | | | | | | | |
| --- | --- | --- | --- | --- | --- | --- | --- | --- | --- |
| **Characteristics** | **Value** |  | **AUC** | **ACC (%)** | **SENS (%)** | **SPEC (%)** | **PPV (%)** | **NPV (%)** | ***p*** |
| Age $\leq$ 50 | yes | I-T | 0.918 [0.825, 0.971] | 82.09 [70.80, 90.39] | 93.33 [77.93, 99.18] | 72.97 [55.88, 86.21] | 73.68 [62.05, 82.74] | 93.10 [77.72, 98.12] | **0.0151** |
|  | no | I-T | 0.794 [0.720, 0.855] | 66.89 [58.77, 74.32] | 90.74 [79.70, 96.92] | 53.61 [43.19, 63.8] | 52.13 [46.38, 57.82] | 91.23 [81.56, 96.07] |  |
| T stage | T1 | I-T | 0.833 [0.754, 0.895] | 71.90 [63.01, 79.69] | 89.19 [74.58, 96.97] | 64.29 [53.08, 74.45] | 52.38 [44.70, 59.95] | 93.10 [84.07, 97.19] | 0.7426 |
|  | T2 | I-T | 0.814 [0.722, 0.886] | 71.13 [61.05, 79.89] | 93.62 [82.46, 98.66] | 50.00 [35.53, 64.47] | 63.77 [56.91, 70.11] | 89.29 [72.93, 96.27] |  |
| ER | positive | I-T | 0.853 [0.789, 0.903] | 82.53 [75.88, 87.98] | 89.23 [79.06, 95.56] | 78.22 [68.90, 85.82] | 72.50 [64.34, 79.39] | 91.86 [84.76, 95.81] | 0.1253 |
|  | negative | I-T | 0.737 [0.596, 0.849] | 67.31 [52.89, 79.67] | 73.68 [48.80, 90.85] | 63.64 [45.12, 79.60] | 53.85 [40.83, 66.36] | 80.77 [65.47, 90.30] |  |
| PR | positive | I-T | 0.839 [0.772, 0.893] | 72.61 [64.93, 79.42] | 96.61 [88.29, 99.59] | 58.16 [47.77, 68.05] | 58.16 [52.28, 63.82] | 96.61 [87.84, 99.12] | 0.6591 |
|  | negative | I-T | 0.811 [0.690, 0.900] | 70.49 [57.43, 81.48] | 80.00 [59.30, 93.17] | 63.89 [46.22, 79.18] | 60.61 [48.85, 71.25] | 82.14 [66.92, 91.27] |  |
| HER2 | positive | I-T | 0.800 [0.677, 0.892] | 66.67 [53.31, 78.31] | 88.89 [65.29, 98.62] | 57.14 [40.96, 72.28] | 47.06 [37.68, 56.65] | 92.31 [75.99, 97.85] | 0.5238 |
|  | negative | I-T | 0.842 [0.776, 0.895] | 74.05 [66.49, 80.69] | 92.42 [83.20, 97.49] | 60.87 [50.14, 70.88] | 62.89 [56.54, 68.81] | 91.80 [82.60, 96.35] |  |
| 95% confidence intervals are included in brackets.  *AUC* area under the receiver operating characteristic curve, *ACC* accuracy, *SENS* sensitivity, *SPEC* specificity, *PPV* positive predict value, *NPV* negative predict value.  *I-T* independent test group, *ER* estrogen receptor, *PR* progesterone receptor, *HER-2* human epidermal growth factor receptor-2, *LNM* lymph node metastasis. | | | | | | | | | |
